# Longitudinal observation of meticillin‐resistant *Staphylococcus pseudintermedius* pulsotypes in six veterinary hospitals in the north‐western United States

**DOI:** 10.1002/vro2.41

**Published:** 2022-08-02

**Authors:** Andrea V. Perkins, Debra C. Sellon, John M. Gay, Eric T. Lofgren, Lisa P. Jones, Margaret A. Davis

**Affiliations:** ^1^ Auburn University College of Veterinary Medicine—Veterinary Clinical Sciences, Vaughan Large Animal Teaching Hospital Auburn Alabama USA; ^2^ Washington State University College of Veterinary Medicine—Veterinary Clinical Sciences Pullman Washington USA; ^3^ Washington State University College of Veterinary Medicine—Paul G. Allen School for Global Health Pullman Washington USA

## Abstract

**Background:**

Meticillin‐resistant *Staphylococcus pseudintermedius* (MRSP) infections in companion animals are increasing and are difficult to treat. Environmental contamination with MRSP in small animal primary care hospitals may pose an exposure risk to animal patients.

**Methods:**

This longitudinal study assessed the genotypic relationships of MRSP isolated from 39 environmental samples collected from six private small animal primary care hospitals, in the north‐eastern United States, between August 2018 and April 2019.

**Results:**

Of the 39 bacterial isolates, 18 unique pulsotypes were identified based on pulsed‐field gel electrophoresis, including six clusters of two or more indistinguishable isolates. Single pulsotypes were frequently detected from multiple hand‐contact and animal‐contact surfaces within a hospital during a single sampling event, but detection of a single pulsotype within the same hospital on subsequent visits was infrequent. However, one pulsotype was recovered from three separate hospitals, which suggests that either MRSP transmission between hospitals may have occurred via people, animals, or fomites or that there was a dominant community strain.

**Conclusions:**

Single strains of MRSP were isolated from various hand‐contact and animal‐contact surfaces within hospitals, indicating the important role of humans, animals and the environment in MRSP transmission. Additionally, the detection of a single strain between hospitals and over time suggests that either MRSP transmission between hospitals may have occurred via people, animals or fomites or that there was a dominant community strain.

## INTRODUCTION

Beta lactam resistance in meticillin‐resistant *Staphylococcus pseudintermedius* (MRSP) isolates results from an altered penicillin binding protein (PBP2a), which is coded by the *mecA* gene.^1^ Meticillin‐resistant *S. pseudintermedius* is an opportunistic commensal of the skin and mucous membranes of an estimated 0%–4% of healthy dogs and is generally transmitted via direct or indirect contact.^2^ Due to increasingly limited antimicrobial therapeutic options^3^ and existing barriers to optimal judicious antimicrobial prescribing practices,^4^ MRSP infections are a growing concern for companion animal veterinarians. Previous studies have reported an MRSP hospital‐level environmental prevalence of 7% in Canada (2010)^5^ and 64% in the United States (2020).^6^

Meticillin‐resistant *S. pseudintermedius* contamination of hospital environments may result in colonisation and infection of companion animal patients exposed to these surfaces. Prudent hospital infection control practices, such as appropriate hand hygiene, cleaning and disinfection, may reduce within‐hospital persistence and transmission potential. To develop infection control measures that break the transmission chain—that reduce further dissemination of antimicrobial resistance genes to people, animals and the environment, thus preserving the efficacy of important antimicrobials—knowledge of MRSP epidemiology in small animal primary care hospitals is needed.

The objective of this study was to increase knowledge about MRSP prevalence and clonality in small animal primary care hospitals and communities. The aims of the study were to describe the presence of MRSP on environmental surfaces in a regional cluster of small animal primary care hospitals, to genotype these isolates and to determine whether environmental contamination by a single strain persisted over time.

## MATERIALS AND METHODS

Recruitment flyers were sent by mail in July 2018 to 54 small animal hospitals in neighbouring cities in two adjacent states in the north‐western United States with a combined human population of approximately 270,493. Hospitals were eligible for inclusion if they identified as mainly providing primary healthcare for dogs and cats. The first six hospitals to respond that met the inclusion criteria were enrolled. The average distance between hospitals was 76.7 km (range: 20.9–160.9 km).

Environmental samples (*n* = 50 per hospital) were collected from a selection of surfaces in each participating hospital monthly for five consecutive months between August 2018 and April 2019 (total samples *n* = 1500). Half of the surfaces sampled were predominantly hand‐contact; half were predominantly animal‐contact (Table 1). Each sample was collected using a new examination glove and electrostatic cloth that was wiped across each surface for approximately 5 s. The cloth was sealed in a sterile plastic bag that was placed in a cooler containing ice packs and then transported directly back to the Washington State University Paul G. Allen School for Global Health laboratory, where 90 ml of tryptic soy broth with 2.5% NaCl was added and then incubated aerobically at 37°C overnight. The transport time following sample collection did not exceed 2 h. Next, samples were streaked onto mannitol salt agar plates (MSA) with oxacillin (2 μg/ml) and incubated for 24–48 h at 37°C. One to three colonies exhibiting typical *Staphylococcus* morphology were sub‐cultured from MSA to Columbia blood agar and incubated at 37°C overnight, and then beta‐haemolytic colonies were coagulase tested. Coagulase‐positive samples were submitted to the Washington Animal Disease and Diagnostic Laboratory for species confirmation using Matrix‐Assisted Laser Desorption Ionisation Time‐of‐Flight Mass Spectrometry. Detection of *mecA* with PCR and pulsed‐field gel electrophoresis (PFGE), including similarity assessment, were carried out according to previously described protocols.^6^, ^7^ In brief, PFGE was carried out according to the published Centers for Disease Control and Prevention unified PFGE protocol for Gram‐positive bacteria.^7^ Slight deviations from this protocol included substitution of the restriction enzyme *Sma*I with *Apa*I and adjusting the run conditions as follows: initial switch time of 1 s, final switch time of 11 s and run time of 18 h, as previously described.^6^ For quality assurance, known *Salmonella enterica* and meticillin‐resistant *Staphylococcus aureus* standards were included in multiple locations on each PFGE gel.

**TABLE 1:** Hand‐contact and animal‐contact surfaces sampled within hospitals

Hand‐contact surfaces	Animal‐contact surfaces
Overhead light handles/knobs	Examination table
Handles of animal enclosures^a^	Ultrasound probe
Computer keyboards/mice	Floor
Light switches	Clipper blades
Ultrasound machine buttons	Muzzles
Clipper handles	Leashes
Counter tops	Inside surfaces of animal enclosures^a^
Door handles	Scale
Drawer/cupboard handles	Surgical restraint straps
Telephone	Pet beds
Scale buttons	Lint roller tape
Otoscope handles	Stethoscope diaphragm
Faucet handles	Gurney
Lead protective vest (outer surface)	Surgery table
Printer/fax machine buttons	Wall^b^
Infusion pump buttons/pole	

^a^ Animal enclosures consisted of cages, kennels, runs and gates.

^b^ Walls were sampled at the level of less than 2 feet from the ground in animal care spaces.

## RESULTS

**FIGURE 1:**
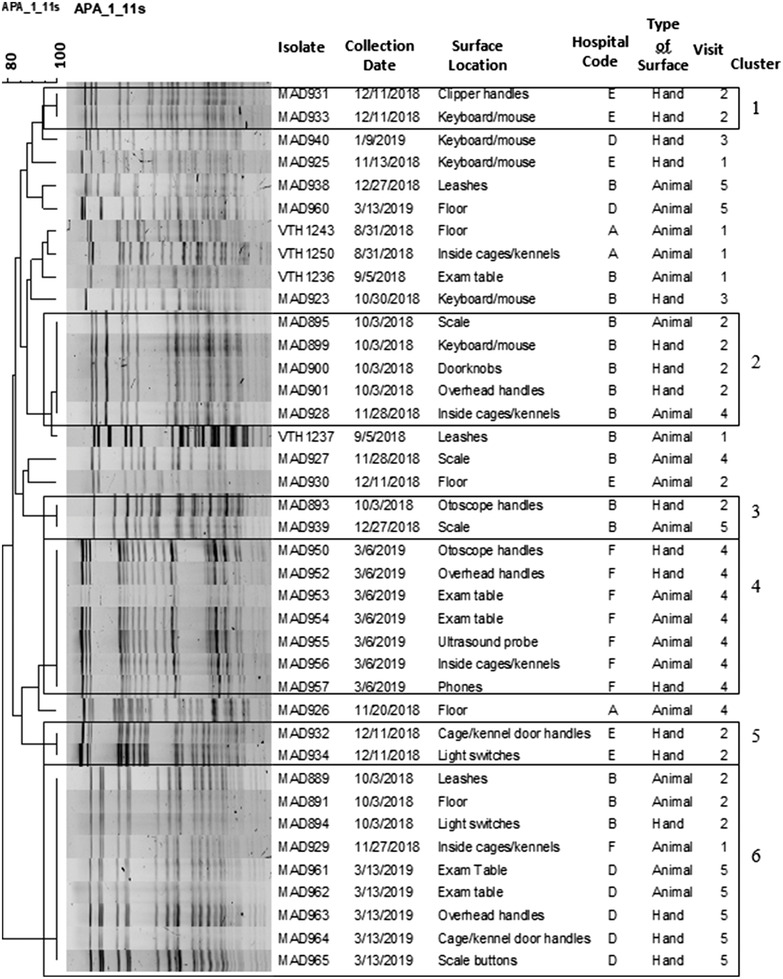
Unweighted pair group method using arithmetic averages cluster analysis of Dice similarities based on pulsed‐field gel electrophoresis of meticillin‐resistant *Staphylococcus pseudintermedius* isolates. Indistinguishable pulsotypes are enclosed in boxes.

Meticillin‐resistant *S. pseudintermedius* was isolated from 3.8% (57/1500) of the samples. Pulsed‐field gel electrophoresis was performed on 39 out of 57 of the isolates; 12 were not available for typing and six were not typeable using the established protocol. Of the 39 isolates, 18 unique pulsotypes were identified, including six unique clusters, a cluster defined as two or more indistinguishable isolates based on PFGE. Isolates with no fragment (band) differences were considered indistinguishable as previously reported.^8^ Three clusters included two isolates (clusters 1, 3 and 5), one cluster included five isolates (cluster 2), one cluster included seven isolates (cluster 4) and one cluster included nine isolates (cluster 6). Clusters 1–5 were recovered from within individual hospitals. Some of the isolates within these clusters were detected on the same visit (clusters 1, 4 and 5), but some were recovered 56 and 85 days apart within the same hospital (hospital B, clusters 2 and 3, respectively). The largest cluster (cluster 6) showed indistinguishable isolates from three hospitals spanning 161 days and 156 km (hospitals B, D and F). All clusters of indistinguishable isolates included at least one isolate recovered from a hand‐contact surface, with two clusters contained isolates recovered from only hand‐contact surfaces (clusters 1 and 5) (Figure 1).

## DISCUSSION

These data show that recovery of indistinguishable MRSP pulsotypes from different environmental surfaces within a hospital during a single sampling event is common. Furthermore, recovery of indistinguishable pulsotypes from both hand‐contact and animal‐contact surfaces during a single sampling event demonstrated that both animals and people are involved in this distribution. Contamination of animal‐contact surfaces is expected considering that MRSP often colonises the skin and mucous membranes, particularly the mouth and perineal area of dogs.^9^, ^10^, ^11^ Because animals were unlikely to directly contact the sampled hand‐contact surfaces (doorknobs, light switches, computer keyboards, etc.) that contaminated healthcare worker hands are a common vehicle for MRSP transmission in these settings is plausible. This finding is consistent with the finding that hand hygiene compliance is generally poor among veterinary healthcare providers.^12^, ^13^ Because it is possible for people to carry MRSP,^3^, ^11^, ^14^, ^15^ proper hand hygiene before and after handling animals or potentially contaminated objects, as recommended by the American Animal Hospital Association and the National Association for State Public Health Veterinarians,^16^, ^17^ should reduce or eliminate hand‐contact transmission. Improving healthcare worker hand hygiene education, as well as the availability and accessibility of essential hand hygiene stations, products and equipment in primary care hospitals, may help reduce the spread of MRSP as well as many other potentially infectious organisms.^18^

In one hospital (hospital B), two unique indistinguishable MRSP pulsotypes were recovered during repeat sampling events (clusters 2 and 3). This suggests that endemic MRSP strains may persist in hospital environments. This spread and persistence may be the result of continual transmission between hands, animals and various environmental surfaces within the hospital or may be the result of a colonised or infected animal being present in hospital B on separate occasions. Animal patients often return to a primary care provider twice or multiple times to treat non‐healing wounds, for post‐surgery re‐checks or to complete a vaccine series, which would provide an opportunity for the same MRSP pulsotype to be reintroduced to a hospital environment. Another possibility is that a hospital employee who cohabits with an MRSP colonised or infected animal repeatedly transmits the agent between their home and their work environment.

Clusters of indistinguishable pulsotypes were frequently recovered from within hospitals during single visits but not on repeat visits. This suggests that after proper cleaning and disinfection, environmental contamination is greatly reduced because MRSP is susceptible to most disinfectants when used according to the manufacturer's instructions.^16^ The pulsotype observed in cluster 6 represented multiple hospitals spanning both time and distance. Unique from other clusters, the pulsotype observed in cluster 6 may represent a successful clone being transmitted within the community. An ad hoc comparison of PFGE profiles of *apa*I‐digested MRSP DNA between isolates in cluster 6 to an international set of 89 MRSP isolates^19^ using multilocus sequence typing did not identify any similarities (Margaret A. Davis, personal communication). A previous cross‐sectional study found that during a single sampling event, different hospitals distributed across a larger geographic range did not share indistinguishable MRSP pulsotypes but that the two closest hospitals had the most closely related pulsotypes.^6^ Our sampling of several hospitals in a tighter geographic cluster provides further support for our findings that small animal primary care hospitals may house persistent endemic strains and that sharing may occur between community hospitals in close proximity through the exchange of people, fomites or animal patients.

This study has several limitations. Due to the small sample size, limited geographic range of participating hospitals and nature of sample acquisition, generalisability is limited. Even so, the findings contribute to a more robust understanding of MRSP transmission dynamics within and between small animal primary care hospitals. Also of note is that because participation was voluntary, infection control behaviour within hospitals may have been altered due to the Hawthorne effect.^20^ If hospital employees increased infection control activities in anticipation of a sampling event, such as enhanced cleaning and disinfection or increased hand hygiene, our survey may have underestimated the true prevalence and diversity of MRSP pulsotypes typically present in such hospital environments. However, the large number of samples collected from multiple hospitals and the longitudinal nature of this study likely mitigated this bias to some extent.

The epidemiology of MRSP and other important multi‐drug‐resistant (MDR) pathogens of concern in small animal primary care hospitals is currently underexplored. Research in this population is important because it is the setting in which most companion animals receive primary medical care. Gaining insight into the epidemiology of MRSP and other MDR pathogens in this population may aid in the innovative design of infection control efforts at the animal–healthcare worker–environment interface. Such efforts may help improve the health and wellness of companion animal patients by preventing within‐hospital pathogen transmission, reduce spread of resistance genes and, ultimately, may help preserve efficacy of antimicrobial therapies important to many species.

## CONFLICTS OF INTEREST

The authors declare they have no conflicts of interest.

## ETHICS STATEMENT

The Washington State University Institutional Review Board approved this research project as exempt.

## AUTHOR CONTRIBUTIONS

All authors contributed substantially to planning, conduct and reporting of the work described. Andrea V. Perkins participated in project conception, planning, design, hospital recruitment, data acquisition, data processing, data interpretation and manuscript writing. Debra C. Sellon, John M. Gay and Eric T. Lofgren participated in conception, planning, design and manuscript writing. Lisa P. Jones participated in project planning, data processing, data interpretation and manuscript writing. Margaret A. Davis participated in project conception, planning, design, hospital recruitment, data interpretation and manuscript writing.

## Data Availability

Data available on request from the authors.
